# Five-year outcome in the copaxone observatory: a nationwide cohort of patients with multiple sclerosis starting treatment with glatiramer acetate in France

**DOI:** 10.1007/s00415-019-09211-5

**Published:** 2019-02-07

**Authors:** Christine Lebrun-Frenay, Antoine Moulignier, Charles Pierrot-Deseilligny, Rabah Benrabah, Thibault Moreau, Catherine Lubetzki, Françoise Monchecourt, the Copaxone Observatory, H. Abboud, F. Abdul Samad, A. Abdulnayef, A. Al Khedr, H. Alchaar, J. Amevigbe, G. Angibaud, O. Anne, M.-S. Artaud-Uriot, G. Ast, J. Augustin, M. Aupy, C. Azais-Vuillemin, M. Bailbe, P. Barbaste, P. Barres, A. Belhadia, R. Benrabah, O. Berets, F.-X. Bergouignan, T. Bierme, F. Bille-Turc, S. Blanc, C. Boisselier, I. Bonnet, J.-P. Borsotti, C. Bossu Van Nieuwenhuyse, C. Bouchard, A. Bouchareine, G. Boudouresques, J.-M. Boulesteix, P. Boulu, B. Bourghol, D. Brassat, A. Bredin, G. Breteau, W. Camu, B. Carlander, W. Casseron, G. Castelnovo, S. Chapuis, J.-P. Chartier, G. Chauplannaz, F. Chaury, C. Cherlet, M. Clanet, P. Clavelou, F. Clerc, R. Colamarino, P. Contis, C. Couratier, M. Coustans, C. Crauser, N. Daluzeau, J.-M. De Bray, T. De Broucker, V. De Burghgraeve, P. De Facq, J. De Sèze, M. Debouverie, G. Defer, I. Degaey, S. Delassaux, J. Delorme, B. Denis, N. Derache-Belpame, O. Dereeper, S.-A. Dereux, F. Derouiche, P. Devos, A.-M. Deyrolle, J. Dib, M. Dib, M. C. Dourneau-Lethiecq, M. Doux, S. Dufourd-Delalande, M. Dujardin, A. Dunac, G. Durand, P. Dussaux, P. Eck, A. Engles, M.-P. Feltin, A. Ferrier, M.-C. Fleury, P. Gaida, O. Gal, A. Gayou-Joyeux, A. Gentil, G. Geraud, J. Gere, J.-C. Getenet, C. Giannesini, E. Giraud, P. Giraud, P. Gras, G. Griffie, C. Gueganno-Roche, A.-M. Guennoc, L. Guilloton, H. Guinot, C. Guiraud-Chaumeil, C. Guiziou, C. Gurau-Vasilescu, N. Guy-Renouil, J.-B. Hamon, O. Heinzlef, C. Hemet-François, J.-L. Henlin, P. Homeyer, P. Hourmant, J.-P. Hurtevent, O. Ille, E. Josien, C. Juhel, M. Kalafat, S. Kelfa-Testut, A. Kopf, C.-P. Kpade, M. Lalisse, D. Laplaud, J. Lapras, J.-L. Larrieu, G. Lavernhe, A. Layet, A. Le Bayon, P. Le Coz, C. Lebrun-Frenay, J. Leche, A. Legout, P. Lejeune, P. Lemarquis, G. Level, J. Lizeretti, D. Locuratolo, M. Logak, P. Louchart, P. Louiset, M. Lubeau, A. Mackowiak, L. Magy, M. Maillet-Vioud, J. Mallecourt, C. Mallecourt, S. Marcel, I. Mari, P. Marrel, J. Maupetit, S. Medjbeur, D. Menard, P. Meynieu, M.-C. Minot-Myhie, B. Montagne, T. Moreau, C. Moreau, A. Moulignier, P. Muh, J.-B. Nkendjuo, A. Nègre, P. Neuschwander, V. Neuville, A. Nibbio, J. Ollier, B. Ondze, J.- C. Ouallet, N. Patte-Karsenti, J. Pelletier, P. Perrotte, D. Pez, C. Pierrot-Deseilligny, J.-C. Pin, S. Pittion-Vouyovitch, C. Popescu, A. Poujois, A. Pouliquen, A. Pouyet, C. Rémy, C. Renglewicz-Destuynder, G. Riche, L. Rieu, C. Robin, A. Robinson, N. Rosey-Dufosse, B. Roualdes, M.-H. Rougie, F. Rouhart, I. Ruggieri, S. Saad, J. Saintarailles, A.-M. Salandini-Roque, M. Salzmann, N. Schmidt, J. Senant, T. Sergeant, V. Seyeux, J. Siboni, O. Simon, S. Stoquart-El Sankari, G. Taurin, S. Thibault-Tanchou, J.-N. Tillier, A. Tougeron, L. Toureille-Borlet, P. Tourniaire, H. Touzani, S. Trefouret, X. Vandamme, J. Vaunaize, F. Viala, U.-C. Viola, J.-M. Visy, M. Vlaicu-Bustuchina, F. Vuillemet, V. Wattier, A. Wavreille, K. Wegener, S. Wiertlewski, C. Zaenker, H. Zephir-Thi, F. Ziegler

**Affiliations:** 1 Service de Neurologie, CRCSEP, Hôpital Pasteur 2, Université Nice Cote d’Azur, CS 51069, 30, voie Romaine, 06001 Nice Cedex 1, France; 2 Fondation Ophtalmologique A. de Rothschild, Paris, France; 3 Département de Neurologie, Hôpital de la Salpêtrière, Paris, France; 4 Centre Hospitalier National d’Ophtalmologie des Quinze-Vingts, Paris, France; 5 Service de Neurologie, Centre Hospitalier Universitaire de Dijon-Bourgogne, Dijon, France; 6 Département des Maladies du Système Nerveux, Hôpital de la Salpêtrière, Paris, France; 7 Teva Santé, La Défense, France

**Keywords:** Relapsing–remitting multiple sclerosis, Disease-modifying treatment, Glatiramer acetate, France, Observational study

## Abstract

The benefits provided by disease-modifying treatments in multiple sclerosis have been demonstrated in clinical trials, but the extent to which they can be extrapolated to everyday care is less clear, as are the long-term benefits of treatment. The objective of this prospective observational cohort study performed in France was to evaluate the effectiveness and safety of glatiramer acetate in patients with relapsing–remitting multiple sclerosis over a 5-year period. All neurologists in France were invited to participate and enroll adult patients starting a first treatment with brand glatiramer acetate 20 mg. Given the observational nature of the study, no fixed study visits were imposed; consultations took place according to the investigator’s normal practice. Occurrence of disease exacerbations and adverse events was documented and neurological disability evaluated with the EDSS at each consultation. Overall, 852 patients were analysable and 269 took glatiramer acetate continuously for 5 years. Median treatment duration was 3.4 years. Principal reasons for discontinuation were inadequate efficacy (38.9%), local tolerability (22.6%) and personal convenience (21.3%). Age, employment status, baseline EDSS score and number of previous exacerbations were variables associated with treatment persistence. The annualised exacerbation rate (5 years) was 0.41 [95% CI 0.39–0.44]; 316 patients (37.2%) remained exacerbation-free throughout. The risk of confirmed disability worsening (5 years) was 43.8% [95% CI 39.9–47.9%]. The most frequent adverse drug reactions were local injection site reactions (584 patients; 68.5%) and systemic immediate post-injection reactions (168 patients; 19.7%). Overall, these findings are consistent with those of previous clinical trials.

**Electronic supplementary material:**

The online version of this article (10.1007/s00415-019-09211-5) contains supplementary material, which is available to authorized users.

## Introduction

Multiple sclerosis is a progressive disabling neurological disease, which affects around 100 000 people in France [1]. It is associated with considerable burden of disease [2] and is the principal cause of non-traumatic irreversible disability in young adults. Over the past 2 decades, a number of disease-modifying treatments (DMTs) for MS have been introduced, which provide a reduction in exacerbation rates and disease activity measured by MRI, together with a possible reduction of disability progression in the medium-term. The first generation of these DMTs were injectable immunomodulatory drugs, namely interferon-β or glatiramer acetate (GA), which have become the mainstay of treatment for patients with relapsing–remitting disease. The first generation of injectable first-line treatments, have been complemented since 2014 (in France) by the first oral first-line therapies.

The benefits provided by DMTs have been clearly demonstrated in randomised clinical trials but the extent to which they can be extrapolated to everyday care is less clear, as are the long-term benefits of treatment. The performance of these treatments in everyday care is of interest both to physicians and to public health authorities who are responsible for allocation of resources for the treatment of chronic diseases. In the light of this uncertainty, several cohort studies have attempted to address this issue. For example, a prospective cohort study including essentially all patients starting interferon-β or GA treatment in the United Kingdom was initiated in the context of a risk-sharing scheme agreed between the health authorities and the manufacturers. After 6 years of follow-up, the observed rate of disability worsening in treated patients was lower than that reported in an untreated historical cohort [3]. Two large patient registries have also been established in Canada which have followed essentially all patients in a provincial catchment area over more than 15 years [4, 5]. Although specific prospective cohorts of patients starting interferon-β treatment for the first time have been established [6], we are not aware of any such prospective cohorts of patients starting GA.

In 2003, at the time of the approval of GA in France, the French health authorities expressed interest in the establishment of an observatory to study the long-term outcome of patients treated with brand GA 20 mg (*Copaxone*^®^) in France. We therefore undertook to establish a nationwide prospective cohort of patients starting GA treatment for the first time and followed for 5 years. The objective of this study was to describe this cohort over the long-term in terms of treatment persistence and modifications, clinical evolution and incidence of adverse drug reactions under real-world conditions of clinical practice.

## Methods

This was a prospective, longitudinal, observational cohort study performed by neurologists treating patients with relapsing–remitting MS in France with GA in everyday conditions of care. Patients were recruited from 2005 to 2008 and each patient was followed up for 5 years or until prematurely lost to follow-up, regardless of whether they continued GA treatment for this period or not. During the period of recruitment, only the original branded *Copaxone* 20 mg formulation of GA was available. Neither the *Copaxone* 40 mg formulation nor generic preparations had yet been marketed.

### Participants

All neurologists in France were invited by mail to participate in the study. Those who accepted were asked to invite all adult patients who were starting treatment with GA for the first time to participate in the study. Both treatment-naïve patients and patients switching from another DMT were eligible.

### Study procedures

Given the observational nature of the study, no fixed study visits were imposed for the purposes of the study. According to the French Health Authority’s guidelines on care for patients with MS [7], patients should see their neurologist at least annually, or more frequently if justified by their clinical evolution [7]. At the inclusion visit, sociodemographic data were documented and the patient’s clinical history taken. Neurological function was evaluated with the Expanded Disability Status Scale (EDSS) [8]. Patients were followed for 5 years after their inclusion visit, or until they were lost to follow-up. At each study visit, any changes in treatment were recorded. Neurological function was re-evaluated with the EDSS [8]. Any exacerbations or adverse drug reactions that had occurred since the previous visit were documented, as were hospitalisations for MS, rounds of corticosteroid treatment and changes in work activity due to MS. All this information was recorded in a case report form for each visit, which was sent to the study coordination centre. Any missing data or inconsistencies were queried with the participating physician before data entry. On-site and telephone monitoring of participating centres was performed episodically to ensure the quality of the data collection.

### Outcome variables

All changes in treatment occurring over the follow-up period were documented. Treatment persistence with GA was determined using Kaplan–Meier actuarial survival analysis, taking the date of the follow-up visit at which no new prescription for GA was issued as the date of discontinuation. Patients who started GA treatment again following a temporary discontinuation were documented but not taken into account in the estimation of persistence.

The annualised exacerbation rate was determined by counting the total number of exacerbations occurring at each study visit between inclusion and study end (or loss to follow-up). These data were introduced into a log-linear model with multiple measures to calculate the number of exacerbations over the follow-up period adjusted for random fluctuations.

EDSS disability scores were only retained if they were measured at least 3 months from an exacerbation. Worsening of disability was only considered confirmed if the EDSS score had increased by at least one point (or 0.5 point if the previous score was ≥ 5.5) since the previous visit and did not decrease at the subsequent follow-up visit at least 3 months later or at any subsequent study visit.

A composite variable of clinical response was also analysed. Clinical response was defined as a patient who had not experienced an exacerbation since the previous visit and whose EDSS disability score had decreased or remained stable since the inclusion visit. The justification for this variable was that follow-up was not systematic and the number of visits at which the EDSS score was not measured was relatively high. In addition, it was not possible to determine at each intermediate visit whether any change in EDSS score was sustained due to the lack of systematic re-evaluation after a fixed interval (3 or 6 months).

Evolution to secondary progressive multiple sclerosis (SPMS) was identified by the neurologist. However, it was verified a posteriori whether these patients fulfilled a standardised criterion for evolution to SPMS, namely no exacerbations in the previous year and an increase of at least one EDSS point over the same period (or of 0.5 point in patients with an EDSS ≥ 5.5 at the beginning of the period).

Adverse drug reactions (ADRs) occurring during the study were noted in the case report form, classified as local reactions, systemic reactions and others. A possible causal role of GA treatment was assessed by the investigator. Only serious ADRs and unexpected ADRS considered to be related to GA treatment were required to be notified by the investigator to the pharmacovigilance department of the study sponsor.

### Statistical analysis

Variables potentially associated with treatment persistence were evaluated in a Cox proportional hazard model. In a first step, univariate analysis was performed to identify all variables associated with persistence. Variables significantly associated at a probability threshold of 0.20 were then entered into a descending stepwise Cox model with only variables retained at a probability threshold of 0.05 being retained for the following round. The variables retained at the end of the procedure were then entered into a final Cox model to generate hazard ratios (HR) with their 95% confidence intervals (95% CI), which were displayed in the form of Forest plots. A similar approach was undertaken to identify variables independently associated with clinical response using multiple logistic regression modelling of a clinical response as a binomial categorical variable.

### Ethics

The study was conducted in conformity with all relevant international and national legislation and guidelines for biomedical research, and with Good Epidemiological Practice. Since the conduct of this study had no influence on the care received by the patients and did not involve any specific study procedures, Ethics Committee approval was not required. All patients participating in the study provided oral consent. The study protocol was submitted and approved by the *Commission Nationale Informatique et Libertés* (CNIL) and the *Comité Consultatif sur le Traitement de l’Information en matière de Recherche dans le domaine de la Santé* (CCTIRS), which are responsible for data protection in France. All patient information in the study database was rendered anonymous.

## Results

### Participants

A total of 412 neurologists agreed to participate, of whom 220 included at least one patient. These corresponded to 103 hospital neurologists, including 44 working in MS reference centres, and 117 community-based neurologists.

**Fig. 1:**
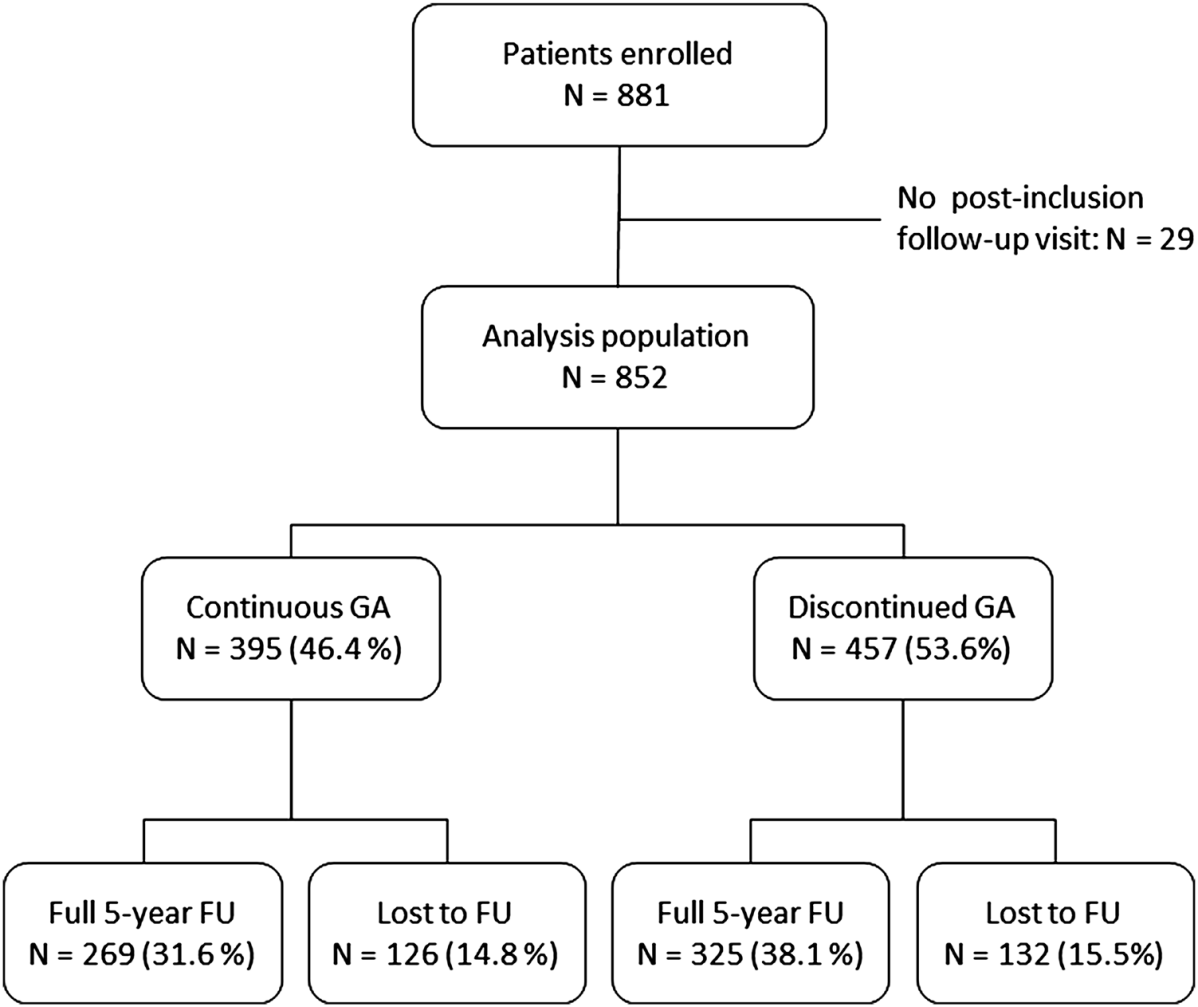
Patient distribution. *GA* glatiramer acetate, *FU* follow-up

The participating neurologists included 881 patients in all, of whom 852 (96.7%) attended at least one documented post-inclusion follow-up visit and were thus retained for the analysis. 280 patients (32.9%) were included in MS reference centres, 176 (20.7%) by other hospital-based neurologists and 396 (46.5%) by community-based neurologists. Of these 852 patients, 258 (30.3%) discontinued the study before the planned 5 years of follow-up had been completed. Of the 594 patients who completed 5 years of follow-up, 269 (45.3%) were still taking GA, the remainder having discontinued or switched. The principal reasons for premature discontinuation were moving house out of the neurologist’s catchment area (*N* = 96) and loss to follow-up (*N* = 53). Three hundred and 95 patients (46.4%) were continually taking GA from inclusion until the end of the study or until loss to follow-up. Of the 457 patients who discontinued GA, 13 had restarted this treatment at the last documented follow-up visit, whereas 225 were receiving another DMT and 219 were untreated. The flow of patients through the study is illustrated in Fig. 1 and the numbers of patients in the study at the end of each 12-month period are provided in Supplementary Table 1.

The sociodemographic and clinical features of the analysis population are presented in Table 1. The mean age of the population was 40 years and 75.8% were women. The mean time between diagnosis and prescription of GA was 8 years although this was very variable (median 6 years; range 0–44 years). The initial presentation was monofocal in around half the cases. In the 2 years prior to inclusion, 10.3% of patients had not experienced an exacerbation whereas 19.3% had experienced three or more. In 67.2% of cases, the patients starting GA who had experienced < 2 relapses in the previous 2 years corresponded principally to those switching from interferon-β for tolerability reasons. Around half had an EDSS score at inclusion > 2 and 6.1% a score > 5. The analysis population contained patients prescribed GA both as a first-line therapy (41.8%) and following a switch from another treatment (58.2%).

**Table 1:** Sociodemographic and clinical characteristics of the analysis population

Variable	All patients *N* = 852	Patients taking GA continuously (*N* = 395)	Patients having stopped GA (*N* = 457)	*P* ^a^	Patients lost to follow-up before 5 years (*N* = 258)	Patients followed for full 5 years (*N* = 594)	*P* ^b^
Age (mean ± SD; years)	39.9 ± 10.6	40.9 ± 10.8	39.1 ± 10.3	0.04	40.1 ± 11.2	39.8 ± 10.3	0.70
Gender (% women)	646 (75.8%)	294 (74.4%)	352 (77.0%)	0.38	189 (73.3%)	457 (78.3%)	0.14
In full-time employment (or student)	394 (46.4%)	206 (52.3%)	188 (41.2%)	0.001	102 (39.8%)	292 (50.0%)	0.006
Not working due to MS	178 (20.9%)	67 (17.0%)	111 (24.3%)	0.009	70 (27.3%)	108 (18.5%)	0.06
Age at first episode (mean ± SD; years)	31.8 ± 9.9	32.8 ± 10.1	31.0 ± 9.6	0.01	32.0 ± 10.3	31.7 ± 9.7	0.68
Time since diagnosis (mean ± SD; years)	8.1 ± 7.5	8.1 ± 7.9	8.1 ± 7.2	0.39	8.1 ± 8.0	8.1 ± 7.3	1.00
Initial presentation							
Optic neuritis	151 (17.9%)	76 (19.5%)	75 (16.6%)	0.28	42 (16.7%)	109 (18.7%)	0.47
Monofocal brainstem symptoms	136 (16.2%)	64 (16.5%)	72 (15.9%)	0.85	37 (14.7%)	99 (17.0%)	0.73
Monofocal spinal cord symptoms	274 (32.5%)	132 (33.9%)	142 (31.3%)	0.46	87 (34.5%)	187 (32.0%)	0.41
Multifocal presentation	264 (31.4%)	108 (27.8%)	156 (34.4%)	0.044	83 (32.9%)	181 (31.0%)	0.24
Paroxysmal symptoms, fatigue, other	17 (2.0%)	9 (2.3%)	8 (1.8%)	0.629	3 (1.2%)	14 (2.4%)	0.25
*Not documented*	10	6	4		6	4	
Exacerbations in the previous 2 years	*N* = 851	*N* = 394	*N* = 457	0.033	*N* = *257*		0.10
0	88 (10.3%)	43 (10.9%)	45 (9.8%)		35 (13.6%)	53 (9.1%)	
1	248 (29.1%)	126 (32.0%)	122 (26.7%)		80 (31.1%)	168 (28.8%)	
2	351 (41.2%)	160 (40.6%)	191 (41.8%)		99 (38.5%)	252 (43.2%)	
≥ 3	164 (19.3%)	65 (16.5%)	99 (21.7%)		43 (16.7%)	121 (20.7%)	
Annual exacerbation rate^c^	0.85	0.81	0.88		0.79		
EDSS^d^	*N* = 716	*N* = 324	*N* = 392		*N* = 209		
Mean score ± SD	2.35 ± 1.66	2.12 ± 1.62	2.54 ± 1.67	< 0.01	2.54 ± 1.73	2.30 ± 1.60	0.05
≤ 1	228 (31.8%)	127 (39.2%)	101 (25.8%)		54 (25.8%)	174 (29.8%)	
1.5 or 2	168 (23.4%)	68 (31.0%)	100 (25.5%)		54 (30.9%)	114 (19.5%)	
2.5 or 3	112 (15.6%)	50 (15.4%)	62 (15.8%)	< 0.01	34 (16.3%)	78 (13.4%)	0.31
3.5 or 4	115 (16.1%)	43 (13.3%)	72 (18.4%)		34 (16.3%)	81 (13.9%)	
4.5 or 5	49 (6.8%)	22 (6.8%)	27 (6.9%)		17 (8.1%)	32 (5.5%)	
> 5	44 (6.1%)	14 (4.3%)	30 (7.7%)		16 (7.7%)	28 (4.8%)	
Previous treatments							
None	356 (41.8%)	191 (48.4%)	165 (36.1%)		118 (45.7%)	238 (40.8%)	
Interferon-β	409 (48.0%)	171 (43.3%)	238 (52.1%)	0.001	112 (43.4%)	297 (50.9%)	0.21
Other	87 (10.2%)	33 (8.4%)	54 (11.8%)		28 (10.9%)	59 (10.1%)	

^a^Comparison between the 395 patients taking GA continuously and the 457 patients discontinuing GA

^b^Comparison between the 258 patients lost to follow-up and the 594 patients followed for 5 years (for both sets of comparisons: *χ*^2^ test for categorical values; Wilcoxon or Student’s *t* test for continuous variables)

^c^Since the number of exacerbations above three was not documented individually, this figure is a lower estimate, assuming that all patients documented as having experienced ≥ 3 exacerbations only experienced precisely three

^d^Last determination before inclusion, at least 3 months distant from an exacerbation

Compared to the analysis population as a whole, the subgroup of 258 patients who did not complete the entire 5-year follow-up as planned were broadly similar (Table 1). The only significant differences observed were that patients lost to follow-up were more likely to have given up work due to their MS and less likely to be in full-time employment.

### Treatment persistence

**Fig. 2:**
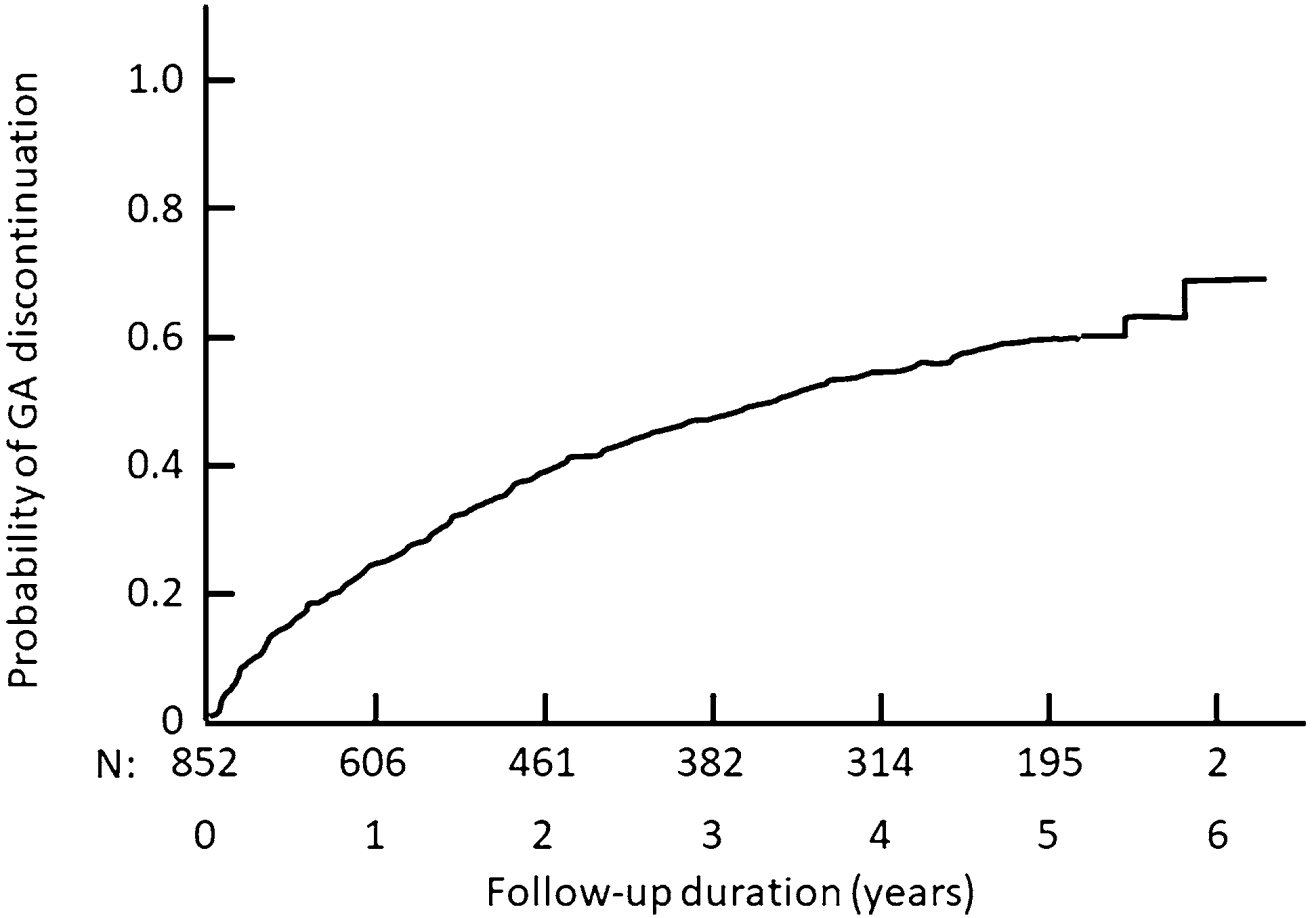
Probability of persistence with AG treatment over the 5-year study period. Data are presented as a Kaplan–Meier survival curve

At the end of the 5-year follow-up period, 260 patients (30.5% of the patients still under observation) had been taking GA continuously throughout the study period. For the 457 patients with a documented discontinuation of GA treatment during the follow-up period, the median duration of treatment estimated using Kaplan–Meier survival analysis was 3.4 years [95% CI 2.9–4.0 years] (Fig. 2). The principal reasons for discontinuing GA were inadequate efficacy and local injection site reactions (Table 2). Only five patients discontinued GA permanently because they became pregnant. Baseline characteristics were compared between the 178 patients discontinuing GA for inadequate efficacy and the 167 discontinuing for tolerance. The only such variable which differed between the two groups was the EDSS score at inclusion, with patients who discontinued for inefficacy having a higher score than those discontinuing for tolerability reasons (2.90 ± 1.68 versus 2.29 ± 1.66; *p* = 0.001). For the 103 patients who discontinued for local injection reactions, the mean treatment duration until discontinuation of GA was shorter than for patients who discontinued for inadequate efficacy (1.1 ± 1.3 years versus 1.8 ± 1.3 years; *p* < 0.0001).

**Table 2:** Patients discontinuing glatiramer acetate: reasons for discontinuation and next-line treatments

	All	No next-line treatment	Interferons	Natalizumab	Fingolimod	Mitoxantrone	Others^b^
Reason for discontinuation^a^	*N* = 457	*N* = 226	*N* = 96	*N* = 81	*N* = 7	*N* = 17	*N* = 30
*Missing data*	*N* = 2	*N* = 2	–	–	–	–	–
Inadequate efficacy	177 (38.9%)	38 (17.0%)	34 (35.4%)	72 (88.9%)	5 (71.4%)	10 (58.8%)	18 (60.0%)
Local tolerability issues	103 (22.6%)	60 (26.8%)	32 (33.3%)	5 (6.2%)	1 (14.3%)	2 (11.8%)	3 (10.0%)
General tolerability issues	71 (15.6%)	48 (21.4%)	19 (19.8%)	–	–	3 (17.6%)	1 (3.3%)
Abnormal laboratory tests	4 (0.9%)	4 (1.8%)	–	–	–	–	–
Occurrence of a serious adverse event	7 (1.5%)	5 (2.2%)	2 (2.1%)	–	–	–	–
Personal convenience	97 (21.3%)	78 (34.8%)	14 (14.6%)	1 (1.2%)	1 (14.3%)	1 (5.9%)	2 (6.7%)
Planned discontinuation	34 (7.5%)	16 (7.1%)	8 (8.3%)	6 (7.4%)	–	1 (5.9%)	3 (10.0%)
Pregnancy or breastfeeding	5 (1.1%)	4 (1.8%)	1 (1.0%)		–	–	–
Other reasons	80 (17.6%)	46 (20.5%)	7 (7.3%)	14 (17.3%)	1 (14.3%)	4 (23.5%)	8 (26.7%)

Data are presented as the number of patients (%)

^a^More than one reason for discontinuation could be provided and these classes are thus not mutually exclusive

^b^These were azathioprine (*N* = 5) and cyclophosphamide (*N* = 25), neither of which are approved for use in MS

Following discontinuation of GA, 231 patients were moved to another disease-modifying treatment, principally an interferon-β or natalizumab. In the remaining 226 patients, no further disease-modifying treatment was documented (Table 2). Patients switched to natalizumab principally did so for inadequate efficacy (72/81 switches). Patients switching to another treatment for tolerability reasons most frequently switched to an interferon-β (53/68 switchers for tolerability). In 38 patients, GA was discontinued for inadequate efficacy but no next-line treatment was documented.

**Fig. 3:**
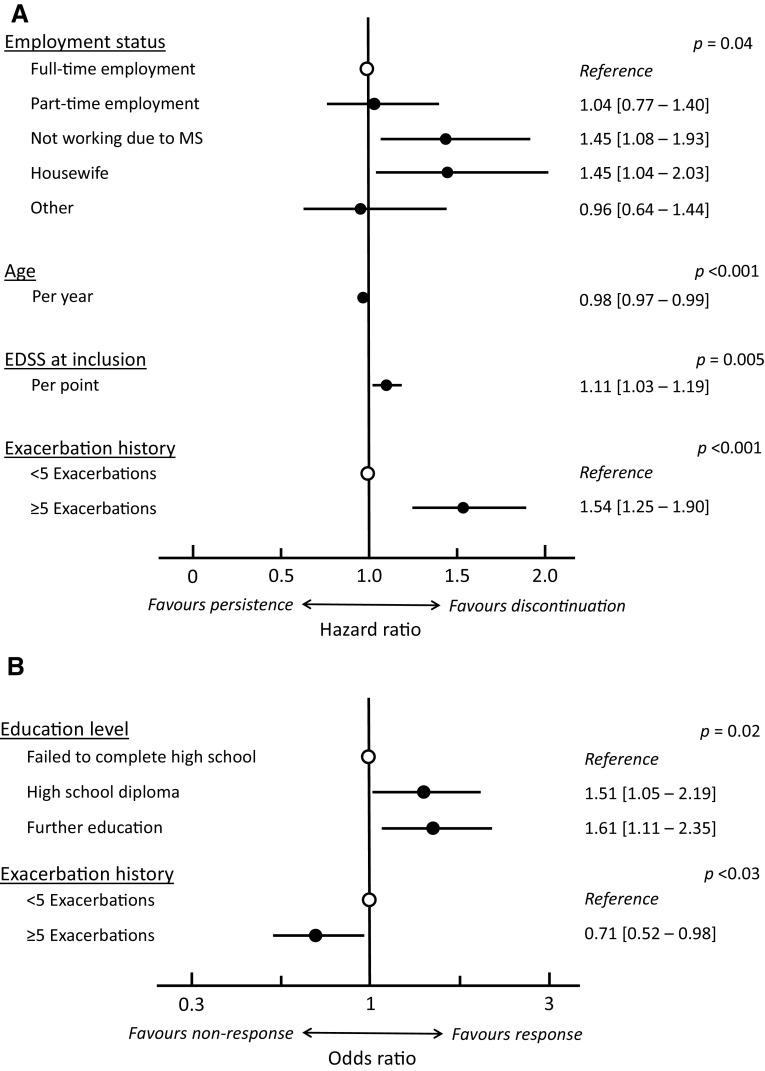
Patient variables independently associated with treatment persistence (**a**; *N* = 715) and with clinical response (**b**; *N* = 661) in patients treated with glatiramer acetate. Data are presented as hazard ratios or odds ratios with their 95% confidence intervals

In univariate analysis, seven variables were significantly (*p* < 0.05) associated with persistence with GA treatment, namely older age when GA treatment was started (*p* = 0.009), being in employment (*p* < 0.001), older age at diagnosis (*p* = 0.007), monofocal presentation at disease onset (*p* = 0.014), a history of < 5 previous exacerbations (*p* < 0.001), lower EDSS score at inclusion (*p* < 0.001) and no previous treatment for MS (*p* < 0.001). Two other variables were identified at the 0.20 probability threshold, namely not living alone (*p* = 0.10) and optic neuritis as first symptom (*p* = 0.098). All these variables were entered into a multivariate Cox proportional hazard model which identified age, employment status, EDSS score at inclusion and the number of previous exacerbations as being independently associated with treatment persistence (Fig. 3a).

### Exacerbations

**Fig. 4:**
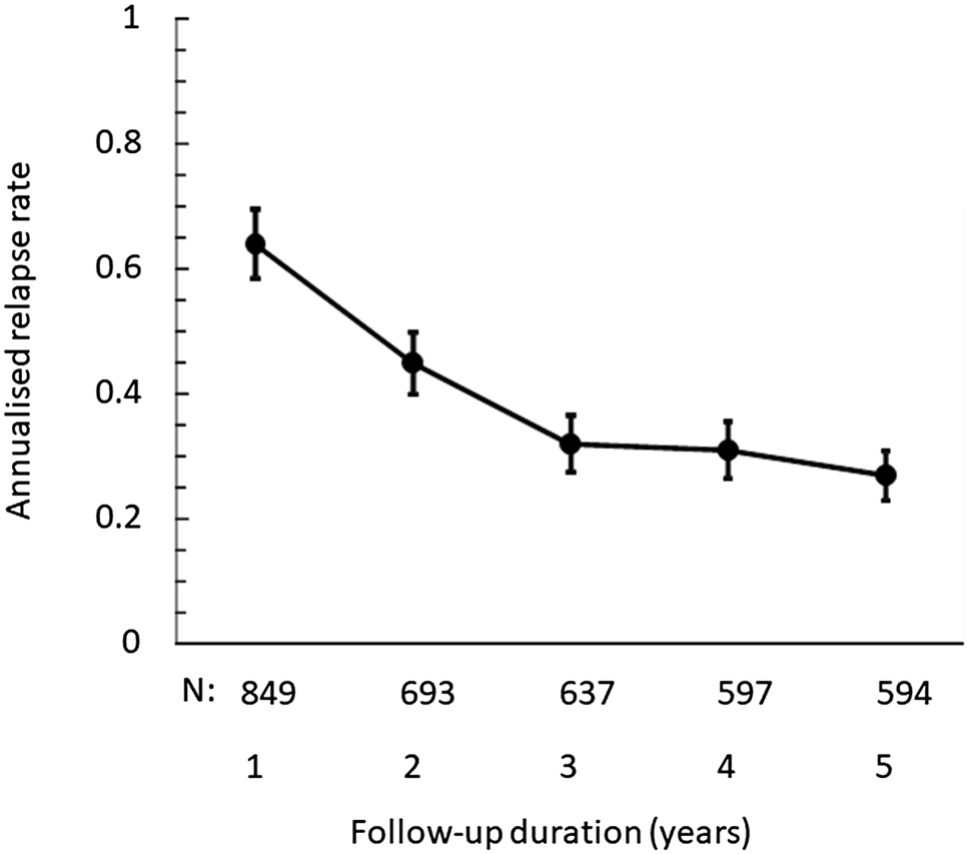
Annualised exacerbation rate over the course of the study

Over the entire follow-up period, the annualised exacerbation rate was 0.41 [95% CI 0.39–0.44] exacerbations/year. This rate decreased progressively from 0.64 [0.59–0.70] exacerbations/year in the first year after initiation of GA to 0.28 [0.24–0.32] exacerbations/year during the fifth year (Fig. 4). Overall, 316 (37.2%) of the patients never presented an exacerbation during the entire follow-up period, whereas 80 patients (9.4%) presented five or more exacerbations. For the 269 patients continuously treated with GA for 5 years, 127 (47.5%; missing data: 10) remained exacerbation-free throughout. In patients who discontinued GA, the on-treatment annualised exacerbation rate was higher and the proportion of patients without exacerbations lower than in patients who continued GA treatment throughout the observation period (*p* < 0.0001; Table 3).

**Table 3:** Clinical outcomes in patients who were treated with GA continuously and in those who discontinued GA treatment

Population	Patients taking GA continuously	Patients discontinuing GA	Patients taking GA continuously	Patients discontinuing GA
Follow-up period	Until last FU visit	Until last FU	At 5 years	At 5 years
	*N* = 395	*N* = 457	*N* = 269	*N* = 325
Annualised exacerbation rate (mean [95% CI])	0.28 [0.23; 0.33]	0.63 [0.56; 0.71]^a^	0.23 [0.19; 0.26]	0.51 [0.46; 0.56]^a^
Exacerbation-free throughout the follow-up period (% [95% CI])	201 (50.9%) [46.0%; 55.8%]	118 (25.8%)^a^ [21.8%; 29.8%]	127 (47.2%) [41.2%; 53.2%]	64 (19.7%)^a^ [15.4%; 24.0%]
Evolution of EDSS score (mean [95% CI])^b^	0.26 [0.14; 0.39]	0.78 [0.63; 0.93]^a^	0.24 [0.09; 0.40]	0.80 [0.62; 0.97]^a^
Patients with no EDSS evolution (% [95% CI])^b^	163/322 (50.6%) [45.2%; 56.1%]	128/387 (33.1%)^a^ [28.4%; 37.8%]	109/226 (48.2%) [41.7%; 54.7%]	79/281 (28.1%)^a^ [22.9%; 33.4%]

^a^Significant difference with patients continually taking GA (*p* < 0.0001; *χ*^2^ test for categorical variables and Student’s *t* test for continuous variables)

^b^These variables could only be assessed in patients evaluated at least 3 months distant from an exacerbation

### Disability

In 136 patients (16.0%), the initial documentation of the EDSS score was not separated from an exacerbation by at least 3 months. For this reason, these patients were excluded from the determination of the EDSS score at inclusion. For the remaining patients, the mean EDSS score at inclusion was 2.4 ± 1.7, being higher in the 457 patients who would subsequently discontinue GA (2.5 ± 1.7) than in the 395 who remained on treatment until their last follow-up visit (2.1 ± 1.6).

**Fig. 5:**
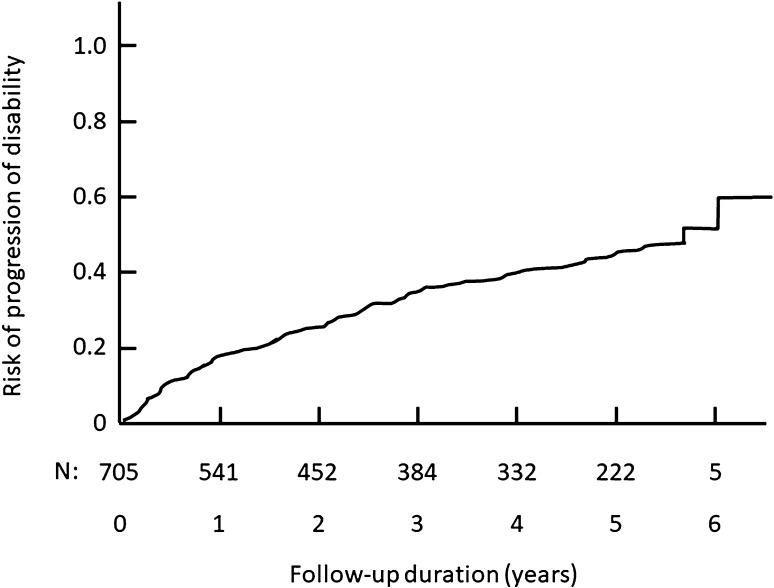
Time to confirmed worsening of disability: Kaplan–Meier survival analysis

At the last study visit, confirmed worsening of disability as defined in the “Methods” was documented in 194 patients (31.9%). According to the Kaplan–Meier survival analysis, the risk of confirmed worsening of disability was 43.8% [95% CI 39.9–47.9%] (Fig. 5). The median time to worsening was not reached during the follow-up period.

In the patients who remained on GA, the mean EDSS score remained relatively unchanged over the course of the study (Table 4) and for 67.1% of the patients the EDSS score was stable or decreased at the end of the 5-year follow-up period. The proportion of patients with a confirmed increase in EDSS score rose gradually over the study. After 5 years, 27.6% of the patients still in the study and taking GA presented an increase in EDSS score ≥ 1 point and 9.7% an increase ≥ 2 points.

**Table 4:** Mean EDSS disability scores and change in EDSS scores since inclusion in patients continually taking GA

Duration of follow-up	*N* ^a^	*n*1^b^	Mean ± SD	Change in EDSS score from inclusion					
				*n*2^c^	Mean ± SD	≤ 0	+ 0.5	+ 1 or 1.5	≥ 2
Inclusion	395	324	2.1 ± 1.6		–	–			
12 months	395	379	1. 9 ± 1.6	315	− 0.10 ± 0.76	258 (81.9%)	25 (7.9%)	27 (8.6%)	5 (1.6%)
24 months	337	308	2.0 ± 1.7	257	0.11 ± 0.93	175 (68.1%)	33 (12.8%)	33 (12.8%)	16 (6.2%)
36 months	308	274	2.1 ± 1.7	231	0.16 ± 1.09	147 (63.6%)	27 (11.7%)	36 (15.6%)	21 (9.1%)
48 months	291	262	2.1 ± 1.7	219	0.22 ± 1.04	137 (62.6%)	20 (9.1%)	41 (18.7%)	21 (9.6%)
60 months	269	243	2.0 ± 1.8	207	0.21 ± 1.11	139 (67.1%)	13 (6.3%)	35 (16.9%)	20 (9.7%)

^a^All patients available for analysis at each annual time-point

^b^Patients whose baseline EDSS score was determined at least 3 months from an exacerbation

^c^Patients whose EDSS scores at baseline and at the follow-up visit were both determined at least 3 months from an exacerbation

Concerning the patients who discontinued GA, the mean EDSS score rose from 2.5 ± 1.7 at inclusion to 3.0 ± 1.8 at the last follow-up visit before stopping GA and to 3.3 ± 2.1 at the last documented follow-up visit. The mean change in EDSS score was 0.5 ± 1.1 during the period of GA treatment and 0.3 ± 1.2 following discontinuation of GA (regardless of whether the patients received a next-line treatment or not), both of these changes being statistically significant (*p* < 0.001; paired Wilcoxon test).

The mean change in EDSS score was lower and the proportion of patients without disability worsening higher in patients who continued GA treatment throughout the observation period compared to those who discontinued treatment (*p* < 0.0001; Table 3).

### Clinical response

A clinical response at 5 years was documented for 318 of 675 patients (47.1%). Response could not be documented for the remaining 177 patients due to incomplete data on exacerbations at certain follow-up visits or on EDSS at the inclusion or final study visit.

In univariate analysis, three variables were significantly (*p* < 0.05) associated with a sustained clinical response, namely higher education level (*p* = 0.020), fewer exacerbations in the 2 years preceding inclusion (*p* = 0.021) and < 5 exacerbations over the entire disease course prior to starting GA (*p* = 0.016) (Supplementary Table 2). Together with six other variables identified at the 0.20 probability threshold (female gender: *p* = 0.057; employment status: *p* = 0.072; shorter time since diagnosis: *p* = 0.053; lower EDSS score at inclusion: *p* = 0.073; no previous DMT therapy: *p* = 0.112; previous interferon-β therapy stopped for inefficacy: *p* = 0.175), these were entered into a multivariate logistic regression analysis, which identified education level and the number of previous exacerbations as being independently associated with clinical response (Fig. 3b).

### Evolution to secondary progressive multiple sclerosis

Over the course of the study, 134 patients (15.8%) were identified by their neurologists as having evolved to a secondary progressive form of MS. For the patients who discontinued GA during the study, this proportion was 20.9% (*N* = 93; data missing for 13 patients). In contrast, 8.1% of patients taking GA for the full 5 years evolved to secondary progressive MS (*N* = 21; data missing: *N* = 10).

Of the patients considered by their neurologist to have evolved to SPMS, 56 (41.8%) fulfilled the objective criterion of no exacerbations in the previous year and an increase of at least one EDSS point. Of the remaining 78 patients, 35 presented relapses in the previous year, and 56 did not have characterised disability worsening of at least one point (some patients fell into both categories).

### Subgroup analysis according to the type of treating neurologist

The principal efficacy outcomes were compared between patients followed in MS reference centres, those treated by other hospital neurologists and in those treated by community neurologists. The data are presented in Supplementary Table 3. No statistically significant differences were observed between the three treatment settings for any of the outcomes evaluated. However, differences between subjective (physician judgement) and objective (decision rules) methods of identifying patients who had evolved to SPMS were observed between the three groups. For neurologists working in reference centres, the two measures provided a similar estimate, whereas the subjective method yielded an over twofold higher estimate than the objective method for the other two physician groups.

### Safety

**Fig. 6:**
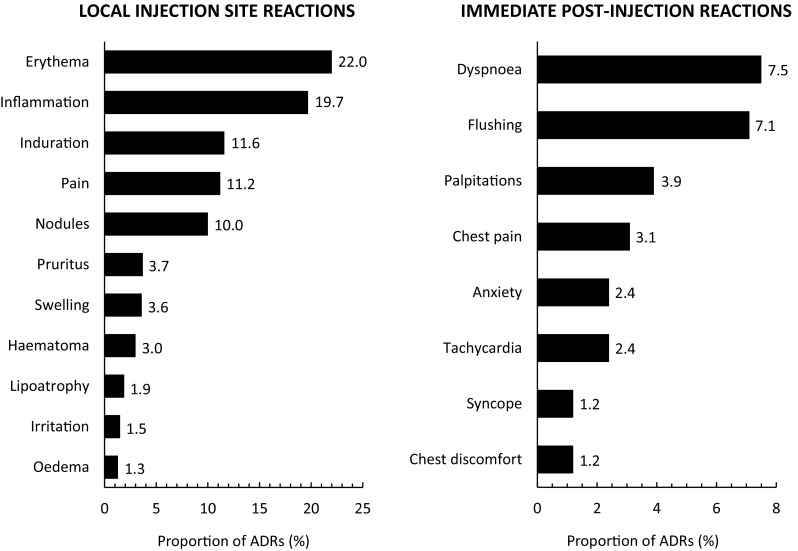
Non-serious adverse drug reactions documented during the study

The AEs reported during the study were principally not serious and were mostly expected. They consisted principally of local injection site reactions (73.6% of non-serious ADRs) reported in 584 patients (68.5%) and systemic immediate post-injection reactions (12.0%) in 168 patients (19.7%). The most frequently reported local reactions were erythema and inflammation and the most frequent systemic reactions were dyspnoea and flushing (Fig. 6).

In addition, 46 serious non-fatal ADRs potentially related to GA were documented in 39 patients (4.6%), notably 18 cases of MS exacerbations (15 patients), four cases of angioedema (three patients) and one case of skin necrosis. Seven patients discontinued GA due to the occurrence of a serious related ADR, in all cases related to a local or systemic injection reaction (anaphylactic reaction, urticaria, injection site pain, Quincke’s œdema, skin necrosis, follicular panniculitis and cutaneous vasculitis). Ten deaths were documented including three suicides. One death was considered to be probably unrelated to GA treatment and the remainder definitely unrelated.

Twenty-two pregnancies occurred in patients taking GA. It should be noted that the prescribing information for GA at the time of the study recommended not using this medication in pregnant women [9]. In most cases, GA was temporarily (*N* = 14) or definitively (*N* = 5) discontinued when the pregnancy was identified. Only three women continued taking GA throughout their pregnancy. Pregnancy outcome was documented in ten cases, of which seven resulted in a term birth of a healthy infant. There was one voluntary pregnancy termination, one spontaneous miscarriage and one in utero death at 9–10 weeks.

## Discussion

This nationwide cohort followed a population of 852 patients with relapsing–remitting MS who initiated treatment with GA over a period of 5 years. Around one-third of these patients were still under GA treatment at the end of the 5-year period. At the last study visit, confirmed worsening of disability was documented in 31.9% of patients, evolution to secondary progressive MS in 13.4%, whereas 37.2% had no documented exacerbation during the study. Clinical outcome was somewhat better in the subgroup of patients continually treated with GA for 5 years (27.6% with confirmed worsening of disability, 8.1% with evolution to SPMS, but 47.5% with no documented exacerbation).

The strengths and limitations of this study largely reflect the naturalistic nature of the study design. The absence of protocol-specified study procedures enabled a large number of patients to be included in a range of settings from specialist MS centres to community neurology practices, and followed up for 5-year. To the extent that the practice of participating neurologists is representative of all MS care in France, which cannot be evaluated, the study provides an accurate picture of actual treatment practices in France. This naturalistic design also brings a certain number of disadvantages for the interpretation of the study. In particular, there is no specified follow-up schedule, leading to a loss of precision for determining the disease course (exacerbation rate, rate of change of EDSS), with a potential risk of bias occurring if patients decide to consult their neurologist only in case of new manifestations of disease. For the same reason, adverse events may be under reported. Given these limitations, the findings should be interpreted conservatively. Apart from infrequent neurologist consultations, another deviation from current practice standards is the absence of information on MRI. At the time the study was designed, there were no consensus standards for MRI in France, and routine use of MRI was essentially restricted to specialist MRI centres. It should be noted that the pivotal clinical trial of GA did not measure MRI outcomes either [10]. The lack of MRI data is a limitation of the study, since it precludes a comprehensive evaluation of residual disease activity. Nonetheless, it is probable that clinical standards have improved over the decade since recruitment into this patient cohort, with more frequent, systematic and comprehensive follow-up of patients. As such, the findings will be of interest as a benchmark for the evolution of standards of care in future long-term prospective naturalistic studies of patients with MS treated with other DMTs.

Clinical outcome following initiation of GA observed in the registry is very close to what has been described previously in the long-term follow-up of the phase III pivotal trial [11]. For example, the annualised exacerbation rate in the fifth year after starting treatment was 0.28 exacerbation/year in our cohort and 0.25 exacerbations/year in the clinical trial extension. The proportion of patients without confirmed worsening of disability was 68% in our study and 58% in the clinical trial extension [11]. These findings suggest that the benefits of GA treatment observed in the context of an interventional clinical trial with a structured patient follow-up protocol in well-selected patients can also be achieved under conditions of everyday care in unselected patients. Similar findings of relatively stable disease (75% of patients without EDSS worsening) have also been reported from a retrospective Spanish cohort of patients treated with GA for at least 5 years [12]. The adverse drug reactions documented over the course of the study were not unexpected for GA [10, 13], corresponding principally to local injection site reactions or systemic immediate post-injection reactions.

A relatively high proportion of patients were lost to follow-up before the end of the 5-year study period (30%). This principally occurred due to the patient no longer consulting the participating neurologist and suggests that measures to ensure continuity of care for these patients with a chronic disease taking long-term DMT therapies would be useful. This relatively high attrition rate does, however, compromise the precision with which we can determine outcome in the overall cohort due to the risk of attrition bias. Another source of missing information concerns the patients for whom the EDSS score at inclusion was measured close to an exacerbation, and thus were excluded from the analysis of evolution of disability.

Thirty percent of patients enrolled into the cohort were treated with GA for the full 5-year period of follow-up. The median treatment duration was 3.4 years. Published reports on-treatment persistence with GA have reported a very wide range of findings, with median treatment durations ranging from 1.7 years [14] to 9.2 years [4], and our study falls within this range. A recent study of over 15,000 patients in the French national MS registry reported a 2-year persistence rate of around 60% for all injectable first-line DMTs [15].

Since 2006, care of patients with MS has been orientated towards multidisciplinary MS Centres, of which there are 18 in France. All these centres participated in our study, although they only recruited one-third of the patients enrolled. This may reflect the relatively recent establishment of the reference studies when the study started. To evaluate the impact of potential differences in standards of care or in patient evaluation (for example, scoring of the EDSS), we performed a subgroup analysis comparing outcome in patients treated in reference centres and in these treated elsewhere. Exacerbation-related outcomes were similar in all three groups. No statistically significant differences in outcome were observed. The largest difference observed between the three groups was the difference between subjective (physician judgement) and objective (decision rules) methods of identifying patients who had evolved to SPMS. Compared to the period of the study, most patients with MS are now expected to be followed in MS reference treatment centres.

The demographic and clinical characteristics of the included population are close to those reported in other relevant populations. Notably, the age at diagnosis and gender ratio are similar to those reported in recent epidemiological surveys of MS in France [16, 17]. Moreover, age and disease duration at start of treatment, gender ratio, EDSS score and pre-treatment exacerbation rates were generally similar to those reported in other cohorts of patients starting GA or interferon-β in Spain [6], Britain [18] and Canada [4]. The rather long disease duration when GA was initiated (8 years) may reflect the fact that the study was initiated shortly after GA was first made available in France, and there was a reservoir of patients who could not be treated by interferon-β awaiting a new treatment. It would be expected that the time between MS onset and treatment initiation would now be much shorter, in accordance with current treatment guidelines.

The principal reasons for treatment discontinuation were inadequate efficacy, local tolerability issues and personal convenience. For patients who discontinued for inadequate efficacy, around half escalated to a second-line treatment, principally natalizumab. The relatively limited recourse to natalizumab (which only became available after the cohort had begun, in 2007) may reflect the requirement that this treatment be provided in MS reference centres only and the fact that most enrolled patients were managed by community-based neurologists. In addition, the study covered the period between the identification of progressive multifocal leucoencephalopathy as an adverse drug reaction to natalizumab and the time when the risk stratification programme for natalizumab had demonstrated its utility; during this period, physicians and patients may have been reluctant to use natalizumab. Patients discontinuing GA due to a tolerability issue were most frequently switched to an interferon-β. Switches to treatments other than natalizumab or interferon-β accounted for < 12% of GA discontinuations. An unanticipated finding was that around half of patients who discontinued GA received no alternative DMT. In part, this may be explained by patients evolving to secondary progressive MS for whom no established DMT existed. These patients did indeed discontinue GA more frequently than patients who remained in a relapsing–remitting phase, but only account for 20% of all discontinuations. It should also be noted that no further treatment options existed for patients who had been treated with an interferon-β before GA, who constituted the majority of patients stopping GA, and for whom natalizumab was not available. It is possible that the imminent arrival of oral therapies encouraged patients to stop GA and await an oral treatment. Fingolimod was the only oral treatment introduced onto the French market during the course of the study (in 2011) as a second-line therapy for patients whose disease remains highly active in spite of adequate treatment with a first-line DMT. Nonetheless, between 2010 and 2014, after the introduction of oral DMTs, around one-third of patients in the national OFSEP patient registry for MS were not prescribed any DMT [15]. A high proportion of untreated patients who discontinue GA or interferon-β without another DMT being introduced has also been reported in a large Canadian cohort [4]. In general, clinical outcomes were worse in patients who discontinued GA than in those taking GA continuously, although it is not possible to determine the direction of any causality.

A number of variables were identified as being associated with a higher probability of treatment discontinuation. These include greater disability (EDSS score) at inclusion, more exacerbations prior to inclusion, younger age at inclusion and not in employment (not working due to MS or being a housewife). Younger age and disability have also been observed to be associated with a greater risk of discontinuation in the Canadian [4] and Catalan [19] prospective cohorts, as well as, for age, in retrospective studies of prescription claims databases [20, 21]. With respect to the association with pre-treatment disease activity, this may be explained by the poorer prognosis of patients with more active or advanced disease [22], leading to a higher probability of discontinuation for poor treatment response. For the association with age, it has been suggested by others that treatment failure may be easier to detect in younger patients [4].

Variables independently associated with clinical response were previous exacerbation history (patients with more exacerbations prior to inclusion had a less favourable prognosis) and educational level. The finding that patients with more active disease prior to inclusion respond less well to DMTs has been observed in a number of other cohorts of patients treated with interferon-β [22–24]. The relationship between response and educational level is more surprising, and we can only hypothesise that lower education levels may be associated with poor adherence to therapy, which in turn, is associated with a lower probability of response.

In conclusion, this study demonstrated that clinical outcome in MS patients treated with GA in everyday clinical care in France was close to that previously demonstrated in interventional clinical trials. Around one-third of patients took GA continuously for 5 years and in general had a favourable outcome. Many patients discontinuing GA did not receive any alternative treatment. This suggests that more specific practice guidelines are needed to guide decisions about discontinuing and switching DMTs; this would be particularly timely given the availability of oral treatments in France and the consequent increase in treatment choice.

## Electronic supplementary material

Below is the link to the electronic supplementary material.

Supplementary material 1 (DOCX 19 KB)
