# Supplementary material for: Five-year outcome in the copaxone observatory: a nationwide cohort of patients with multiple sclerosis starting treatment with glatiramer acetate in France
Source: J Neurol. 2019 Feb 7;266(4):888–901. doi: 10.1007/s00415-019-09211-5 (PMC6420902; doi:10.1007/s00415-019-09211-5)
Supplement: Supplementary file 1 — Supplementary material 1 (DOCX 19 KB) [file 415_2019_9211_MOESM1_ESM.docx]

# SUPPLEMENTARY MATERIAL

**Supplementary Table 1.** Patient disposition at the end of each 12-month period during the study.

|  | Inclusion N=852 | 12 Mo N=852 | 24 Mo N=852 | 36 Mo N=852 | 48 Mo N=852 | 60 Mo N=852 |
| --- | --- | --- | --- | --- | --- | --- |
|  | 852 | 852 | 852 | 852 | 852 | 852 |
| GA ongoing | 852 (100.0%) | 641 (75.2%) | 472 (55.4%) | 386 (45.3%) | 325 (38.1%) | 269 (31.6%) |
| GA stopped - Other DMD ongoing | 0 (0.0%) | 96 (11.3%) | 139 (16.3%) | 162 (19.0%) | 170 (20.0%) | 171 (20.1%) |
| GA stopped - No DMD ongoing | 0 (0.0%) | 112 (13.1%) | 122 (14.3%) | 137 (16.1%) | 143 (16.8%) | 144 (16.9%) |
| GA restarted | 0 (0.0%) | 3 (0.4%) | 5 (0.6%) | 4 (0.5%) | 5 (0.6%) | 10 (1.2%) |
| Patient LFU - GA ongoing | 0 (0.0%) | 0 (0.0%) | 58 (6.8%) | 87 (10.2%) | 104 (12.2%) | 126 (14.8%) |
| Patient LFU - GA restarted and ongoing | 0 (0.0%) | 0 (0.0%) | 0 (0.0%) | 1 (0.1%) | 2 (0.2%) | 3 (0.4%) |
| Patient LFU - GA stopped - Other DMD ongoing | 0 (0.0%) | 0 (0.0%) | 18 (2.1%) | 26 (3.1%) | 40 (4.7%) | 54 (6.3%) |
| Patient LFU - GA stopped - No DMD ongoing | 0 (0.0%) | 0 (0.0%) | 38 (4.5%) | 49 (5.8%) | 63 (7.4%) | 75 (8.8%) |

DMD: disease-modifying treatment; GA: glatiramer acetate; LFU: lost to follow-up.

**Supplementary Table 2.** Factors associated with clinical response identified in univariate analysis.

| Subgroup | N | Response rate | Odds ratio | P value |
| --- | --- | --- | --- | --- |
| *Gender* |  |  |  | 0.057 |
| Men | 161 | 65 (40.4%) | 1.00 |  |
| Women | 520 | 253 (49.8%) | 1.43 [1.00; 2.05] |  |
| *Education level* |  |  |  | 0.020 |
| Primary | 266 | 108 (40.6%) | 1.00 |  |
| Secondary | 204 | 104 (51.0%) | 1.52 [1.05; 2.20] |  |
| Tertiary | 191 | 100 (52.4%) | 1.61 [1.11; 2.34] |  |
| *Employment status* |  |  |  | 0.072 |
| Full-time employment (incl. students) | 313 | 155 (49.5%) | 1.00 |  |
| Part-time employment | 101 | 55 (54.5%) | 1.22 [0.78; 1.91] |  |
| Invalidity status | 142 | 56 (39.4%) | 0.66 [0.44; 0.99] |  |
| Housewife | 65 | 32 (49.2%) | 0.99 [0.58; 1.69] |  |
| Others (children, retired, job-seeking) | 54 | 20 (37.0%) | 0.60 [0.33; 1.09] |  |
| *Exacerbations in the previous two years* |  |  |  | 0.021 |
| 0 | 77 | 44 (57.1%) | 1.00 |  |
| 1 | 210 | 104 (49.5%) | 0.74 [0.43; 1.25] |  |
| 2 | 267 | 120 (44.9%) | 0.61 [0.37; 1.02] |  |
| ≥3 | 121 | 50 (41.3%) | 0.53 [0.30; 0.94] |  |
| *Total number of previous exacerbations before GA start* |  |  |  | 0.016 |
| <5 | 383 | 196 (51.2%) | 1.00 |  |
| ≥5 | 292 | 122 (41.8%) | 0.68 [0.50; 0.93] |  |
| *Time since diagnosis* |  |  |  | 0.053 |
| Continuous - by year | 675 | - | 0.98 [0.96; 1.00] |  |
| EDSS score at inclusion |  |  |  | 0.073 |
| Continuous - by grade | 675 | - | 0.92 [0.84; 1.01] |  |
| *Previous DMT therapy* |  |  |  | 0.112 |
| None | 254 | 132 (52.0%) | 1.00 |  |
| Interferon-β | 346 | 150 (43.4%) | 0.71 [0.51; 0.98] |  |
| Other | 75 | 36 (48.0%) | 0.85 [0.51; 1.43] |  |
| *Previous IFN stopped for inefficacy* |  |  |  | 0.175 |
| No | 565 | 273 (48.3%) | 1.00 |  |
| Yes | 110 | 45 (40.9%) | 0.74 [0.49; 1.12] |  |

DMD: disease-modifying treatment; EDSS: extended disability status score; IFN: interferon-β.

**Supplementary Table 3.** Clinical outcome as a function of the type of treating neurologist.

| Outcome variable | MS Reference centre N = 280 | Other hospital neurologist N = 176 | Community neurologist N = 396 |
| --- | --- | --- | --- |
| Annualised exacerbation rate | 35.1% [29.7 – 40.8] | 36.6% [29.8 – 43.9] | 39.0% [34.3 – 43.9] |
| Exacerbation free | 0.42 [0.39 – 0.48] | 0.41 [0.36 – 0.48] | 0.41 [0.38 – 0.45] |
| Evolution to SPMS (physician)^a^ | 12.6% [9.2 – 17.0] | 16.6% [11.8 – 22.8] | 17.8% [14.3 – 21.9 |
| Evolution to SPMS (algorithm)^b^ | 9.8% [6.6 – 14.3] | 6.2% [3.3 – 11.3] | 7.4% [5.0 – 10.8 |
| Worsening of disability (EDSS) | 38.7% [32.6 – 45.1] | 46.2% [38.2 – 54.3] | 38.8% [33.6 – 44.3] |

The analysis was performed on the total cohort of patients enrolled and the evaluation period was from inclusion until five years or loss to follow up. Data are presented within their 95% confidence intervals.

EDSS: extended disability status score; SPMS: secondary progressive multiple sclerosis.

^a^According to the physician’s judgement; ^b^No exacerbation in previous year and increase in EDSS ≥1 point.
